# Membrane trafficking and mitochondrial abnormalities precede subunit c deposition in a cerebellar cell model of juvenile neuronal ceroid lipofuscinosis

**DOI:** 10.1186/1471-2202-5-57

**Published:** 2004-12-10

**Authors:** Elisa Fossale, Pavlina Wolf, Janice A Espinola, Tanya Lubicz-Nawrocka, Allison M Teed, Hanlin Gao, Dorotea Rigamonti, Elena Cattaneo, Marcy E MacDonald, Susan L Cotman

**Affiliations:** 1 Molecular Neurogenetics Unit of Department of Neurology and Center for Human Genetic Research, Massachusetts General Hospital, Charlestown, MA, USA; 2 Department of Pharmacological Sciences and Center of Excellence on Neurodegenerative Diseases, University of Milano, Milan, Italy

## Abstract

**Background:**

JNCL is a recessively inherited, childhood-onset neurodegenerative disease most-commonly caused by a ~1 kb *CLN3 *mutation. The resulting loss of battenin activity leads to deposition of mitochondrial ATP synthase, subunit c and a specific loss of CNS neurons. We previously generated *Cln3*^Δex7/8 ^knock-in mice, which replicate the common JNCL mutation, express mutant battenin and display JNCL-like pathology.

**Results:**

To elucidate the consequences of the common JNCL mutation in neuronal cells, we used P4 knock-in mouse cerebella to establish conditionally immortalized Cb*Cln3 *wild-type, heterozygous, and homozygous neuronal precursor cell lines, which can be differentiated into MAP-2 and NeuN-positive, neuron-like cells. Homozygous Cb*Cln3*^Δex7/8 ^precursor cells express low levels of mutant battenin and, when aged at confluency, accumulate ATPase subunit c. Recessive phenotypes are also observed at sub-confluent growth; cathepsin D transport and processing are altered, although enzyme activity is not significantly affected, lysosomal size and distribution are altered, and endocytosis is reduced. In addition, mitochondria are abnormally elongated, cellular ATP levels are decreased, and survival following oxidative stress is reduced.

**Conclusions:**

These findings reveal that battenin is required for intracellular membrane trafficking and mitochondrial function. Moreover, these deficiencies are likely to be early events in the JNCL disease process and may particularly impact neuronal survival.

## Background

Juvenile neuronal ceroid lipofuscinosis (JNCL), or Batten disease, is a recessively inherited childhood-onset neurodegenerative disorder characterized by progressive blindness, seizures, motor and cognitive decline, and early death [1]. The primary genetic defect (>80% disease chromosomes) leading to JNCL is a 1.02 kb genomic DNA deletion in the *CLN3 *gene, which eliminates exons 7 and 8 and surrounding intronic DNA, predicting a non-functional protein product [2].

The pathological hallmark of JNCL is autofluorescent ceroid lipofuscin deposits within autolysosomes that are enriched in subunit c of the mitochondrial ATP synthase complex [3-5]. Remarkably, these deposits are not only found in CNS neurons but are also abundant in non-neuronal cells outside of the nervous system. The relationship of subunit c deposits to the JNCL disease process, and the underlying reason for the neuronal specificity of the disease remain poorly understood.

The *CLN3*-encoded protein (battenin, also called CLN3 or cln3 p) is a highly conserved, ubiquitously expressed, multi-pass membrane protein [6] that localizes to the lysosome and other vesicular compartments [7-9]. Battenin function remains to be elucidated, although studies of *btn1*, the yeast *CLN3 *ortholog, have implicated battenin in lysosomal pH homeostasis and amino acid transport [10,11].

To explore JNCL pathogenesis and battenin function, we previously generated a genetically precise JNCL mouse model. *Cln3*^Δex7/8 ^knock-in mice harbor the ~1 kb common JNCL mutation and express a non-truncated mutant battenin isoform that is detectable with antibodies recognizing C-terminal epitopes. Homozygous *Cln3*^Δex7/8 ^knock-in mice exhibit a progressive JNCL-like disease, with perinatal onset of subunit c deposition in many cell types and later onset of neuronal dysfunction and behavioral deficits [12]. These findings suggest that the major JNCL defect leads to abnormal turnover of mitochondrial subunit c, in a manner that selectively compromises CNS neurons.

Currently, there is no suitable neuronal cell system to investigate the impact of the common JNCL mutation on biological processes. Therefore, we have established cerebellar neuronal precursor cell lines from *Cln3*^Δex7/8 ^knock-in mice. Homozygous Cb*Cln3*^Δex7/8 ^cells exhibit pathological hallmarks of the disease, and a survey of membrane organelles revealed membrane trafficking defects and mitochondrial dysfunction in homozygous mutant Cb*Cln3*^Δex7/8 ^cells.

## Results

### Generation of a genetically precise cerebellar JNCL cell model

**Figure 1:**
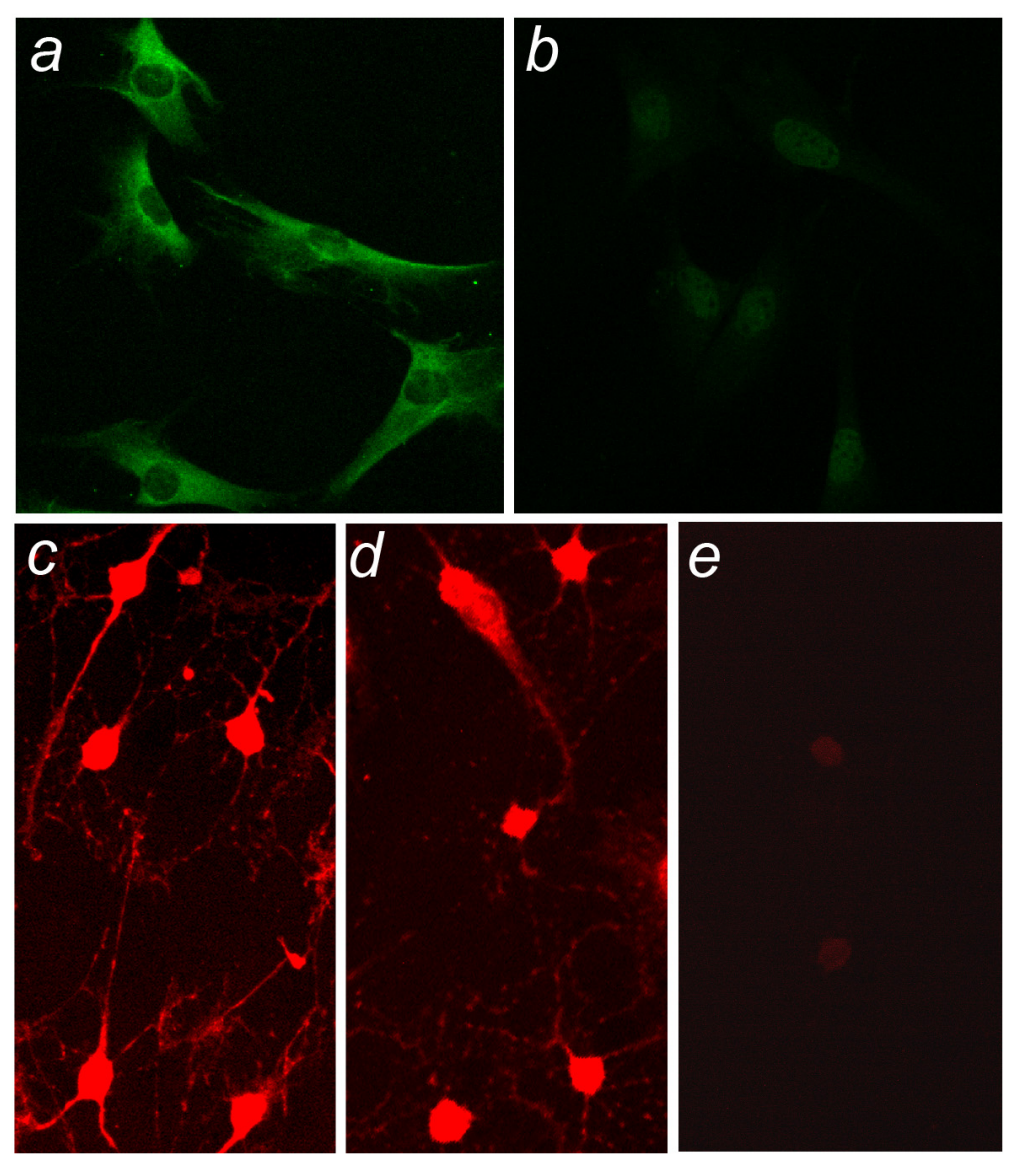
**Neuronal marker expression in Cb*Cln3*^+/+ ^cells **Characterization of Cb*Cln3*^+/+ ^cells by immunofluorescence with marker antibodies is shown. Cb*Cln3*^+/+ ^precursors exhibit nestin expression (a) but not GFAP expression (b), consistent with a neuronal precursor identity. Upon stimulation with a differentiation cocktail (see Methods), Cb*Cln3*^+/+ ^cells achieved neuron-like morphology, with rounded cell bodies and extension of processes, and MAP2 (c) and NeuN (d) expression was increased. Cb*Cln3*^+/+ ^cells are negative for the Purkinje neuron marker calbindin (e). Cb*Cln3*^+/Δex7/8 ^and Cb*Cln3*^Δex7/8/Δex7/8 ^cell lines exhibited identical marker immunofluorescence results. a, b) 20 × magnification; c, d, e) 40 × magnification.

To generate a precise genetic, neuron-derived JNCL cell culture system, we immortalized granule neurons cultured from postnatal day 4 (P4) cerebella of homozygous and heterozygous *Cln3*^Δex7/8 ^knock-in mice, and wild-type littermates. Primary cell cultures enriched for granule neurons were transduced with retroviral vector bearing a selection cassette and temperature-sensitive tsA58 SV40 large T antigen. Growth in G418 containing medium at the permissive temperature (33°C) allowed for selection and isolation of multiple clonal nestin-positive (Fig. 1a), and GFAP-negative (Fig. 1b), cell lines for each genotype. No genotype specific differences were observed in cellular morphology or doubling time (~46 hours) (data not shown). As expected, SV40 large T antigen expression was rapidly lost and cell division ceased when cells were shifted to the non-permissive temperature (39°C) (data not shown). Upon addition of neuronal differentiation cocktail, precursor cells became neuron-like in morphology and exhibited decreased nestin expression (data not shown) and increased MAP2 and NeuN expression (Fig. 1c,1d), but not expression of the Purkinje marker, calbindin (Fig. 1e).

### Homozygous Cb*Cln3*^Δex7/8 ^cells express mutant battenin and display JNCL-like pathology

**Figure 2:**
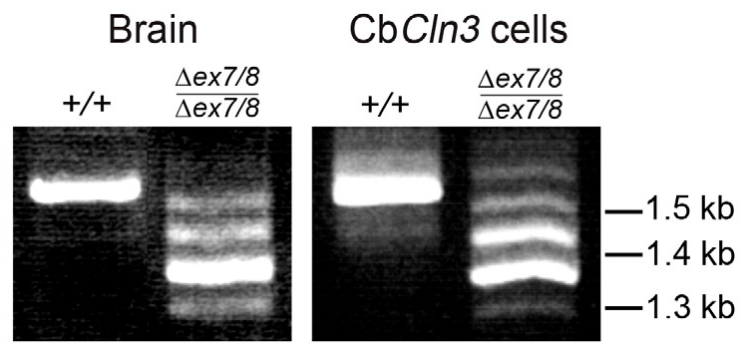
**RT-PCR of Cln3 mRNA in wild-type and homozygous Cb*Cln3*^Δex7/8 ^cells ***Cln3 *Exon1-forward, Exon 15-reverse RT-PCR products are shown, from total wild-type (+/+) or homozygous mutant (Δex7/8/Δex7/8) brain and cell line RNA. Brain and cell line RT-PCR reaction products had identical band patterns on ethidium-bromide stained agarose gels. Wild-type RT-PCR product was a single ~1.6 kb band and mutant products were ~1.6, ~1.5, ~1.4, ~1.35, and ~1.3 kb, representing multiple mutant splice variants.

**Figure 3:**
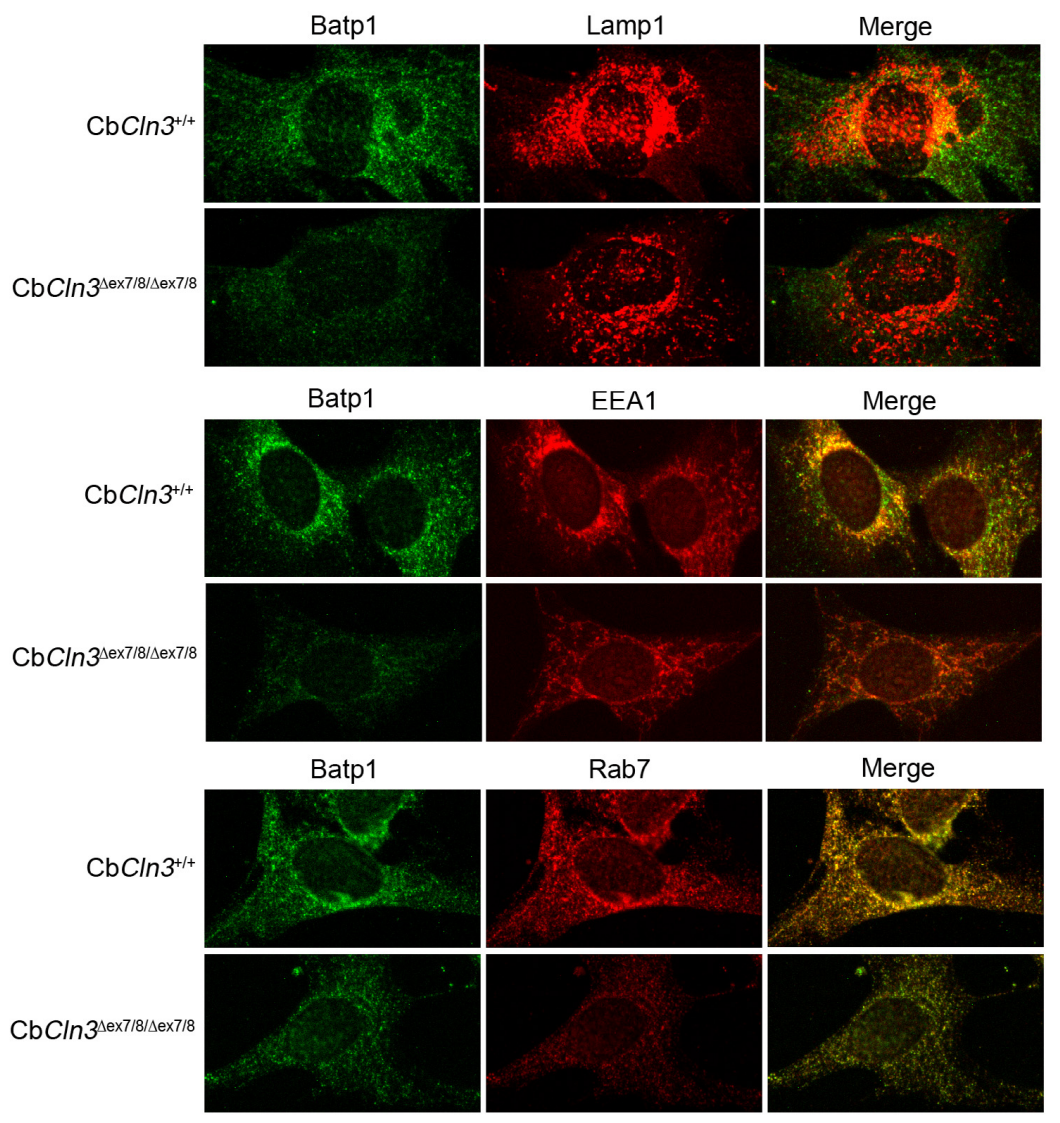
**Battenin and lysosomal and endosomal marker co-staining in wild-type and homozygous Cb*Cln3*^Δex7/8 ^cerebellar precursor cells **Batp1 immunostaining of wild-type (Cb*Cln3*^+/+^) and homozygous mutant (Cb*Cln3*^Δex7/8/Δex7/8^) cerebellar precursor cells is shown, with co-staining for lysosomes (Lamp 1), early endosomes (EEA1), and late endosomes (Rab7). Significant overlap of Batp1 signal (red) with EEA1 (green, middle panels) and Rab7 (green, bottom panels) can be seen as yellow when the two channels are merged (Merge). The degree of Batp1 overlap is greatest with Rab7. Only limited overlap between Batp1 (red) and Lamp 1 (green, top panels) can be seen. Batp1 signal in homozygous Cb*Cln3*^Δex7/8 ^cells is significantly reduced, but significant overlap with EEA1 and Rab7, and very little Lamp 1 overlap, can be seen as yellow in the respective merged panels. Notably, Lamp 1 and EEA1 localization appear altered, and Rab7 staining was frequently less intense in homozygous Cb*Cln3*^Δex7/8 ^cells. Wild-type and homozygous Cb*Cln3*^Δex7/8 ^confocal images were captured with identical exposure settings. 60 × magnification.

Homozygous Cb *Cln3*^Δex7/8 ^cells were first examined for JNCL-like characteristics. Homozygous *Cln3*^Δex7/8 ^knock-in mice express multiple *Cln3 *mRNA splice variants and mutant battenin protein that is detectable by batp1 antibody recognizing C-terminal epitopes [12]. To assess this molecular phenotype in Cb*Cln3*^Δex7/8 ^cells, RT-PCR and anti-battenin (batp1) immunostaining were performed. As shown in Figure 2, *Cln3 *mRNA isoforms in wild-type and homozygous cells were similar to those observed in total RNA isolated from wild-type or homozygous *Cln3*^Δex7/8 ^knock-in brain, respectively (Fig. 2). In addition, batp1 immunostaining detected mutant battenin product in homozygous Cb*Cln3*^Δex7/8 ^cells, in a similar albeit reduced cytoplasmic, vesicular staining pattern as that seen in wild-type cells. Batp1 signal exhibited some overlap with the lysosomal marker, Lamp1, but had more significant overlap with early endosome antigen 1 (EEA1) and the late endosomal marker, Rab7 (Fig. 3). Only limited overlap was observed with recycling endosomes, as determined by transferrin receptor co-staining (data not shown). Intriguingly, Lamp1 and EEA1 immunocytochemical distribution were altered in homozygous Cb*Cln3*^Δex7/8 ^cells, with less perinuclear clustering than in wild-type cells, and Rab7 staining was frequently less intense in homozygous Cb*Cln3*^Δex7/8 ^cells (Fig. 3). Heterozygous Cb*Cln3*^Δex7/8 ^cells contained a mixture of *Cln3 *mRNA products from both the wild-type allele and the mutant allele, and batp1 signal was similar to that seen in wild-type cells (data not shown).

**Figure 4:**
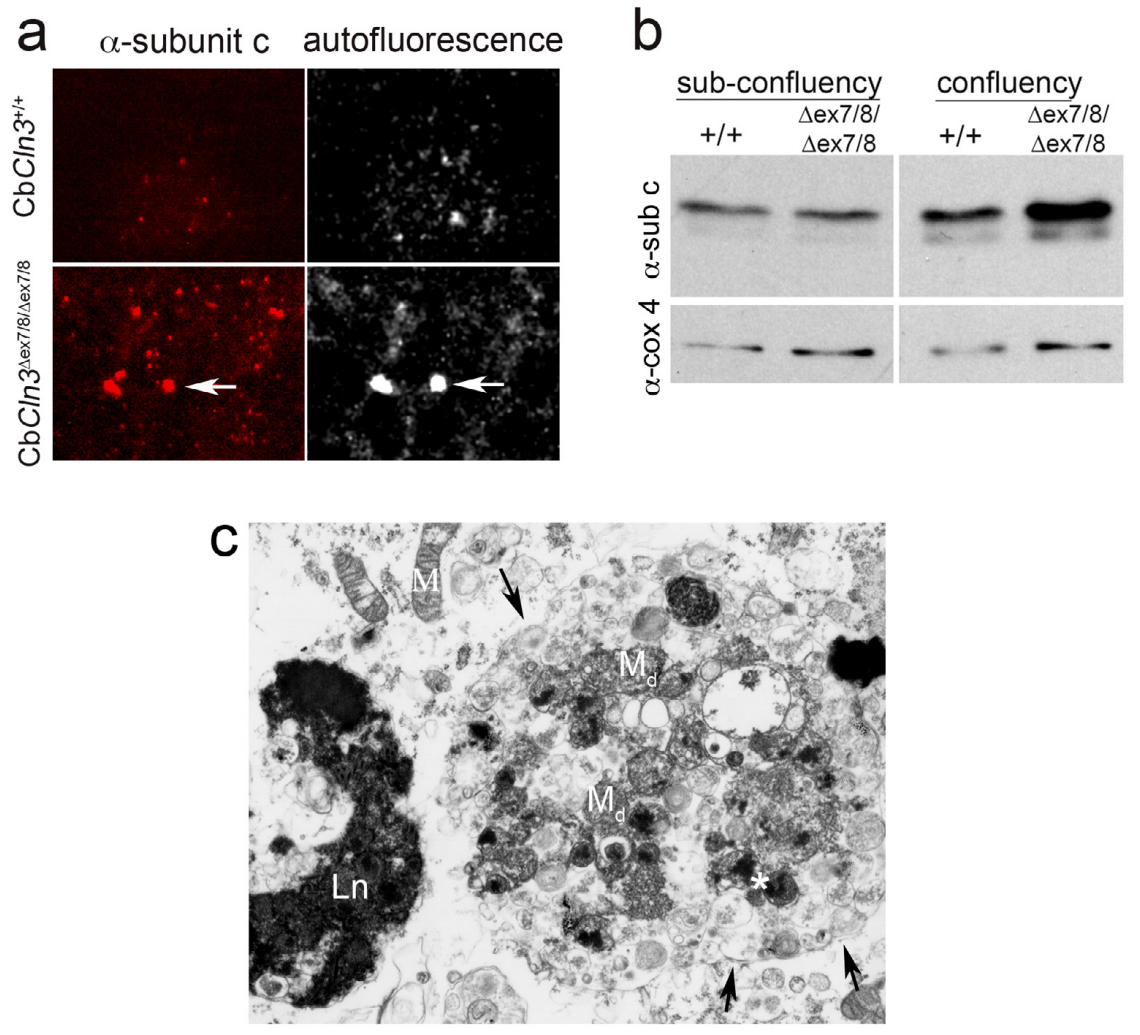
**Subunit c accumulation in homozygous Cb*Cln3*^Δex7/8 ^cerebellar precursor cells **a. Subunit c immunostaining and autofluorescence of 7-day confluency-aged wild-type and homozygous Cb*Cln3*^Δex7/8 ^cells is shown. Wild-type cultures (Cb*Cln3*^+/+^) exhibited limited subunit c immunostaining and autofluorescence. However, Cb*Cln3*^Δex7/8/Δex7/8 ^cells contained numerous subunit c puncta. Autofluorescence (7 days AF) was also significantly elevated (right panels), although limited overlap with subunit c puncta was observed (arrows). 40 × magnification. b. Immunoblot analysis of subunit c protein at sub-confluency or 7-day confluency incubation is shown. Total protein extracts from sub-confluency wild-type (+/+) and homozygous mutant (Δex7/8/Δex7/8) cultures contained approximately equal levels of subunit c protein (α-sub c). 7-day confluency extract from homozygous Cb*Cln3*^Δex7/8 ^cells (Δex7/8/Δex7/8) had elevated levels of subunit c protein (~1.5X), relative to wild-type extract (+/+). Protein levels were normalized to cytochrome c oxidase subunit IV (α-cox4). c. TEM analysis of inclusions in 7-day confluency-aged homozygous Cb*Cln3*^Δex7/8 ^cells is shown. A large autophagosome contained by double membrane (arrows) is filled with degenerating mitochondria (M_d_), electron dense cores (left and right of *) and other smaller vesicular structures. A large electron-dense inclusion, with a lipofuscin (Ln) appearance, is also present. M, mitochondria. 10,000 × magnification.

During sub-confluent growth conditions, neither wild-type nor homozygous Cb*Cln3*^Δex7/8 ^cells displayed autofluorescence or subunit c inclusion formation (data not shown). However, when cells were aged at confluency (3+ days post-confluency), homozygous Cb*Cln3*^Δex7/8 ^cellular subunit c levels were elevated beyond normal wild-type levels by immunostaining (Fig. 4a) and immunoblot analysis (Fig. 4b). Autofluorescent signal sometimes overlapped with subunit c signal, but also was elevated more diffusely (Fig. 4a). Moreover, although multilamellar "fingerprint" profiles were not detected, confluency-aged homozygous Cb*Cln3*^Δex7/8 ^cells displayed numerous ultrastructural abnormalities including electron dense inclusions characteristic of lipofuscin and large autophagosomes that contained dense core structures, degenerating mitochondria, and many smaller vesicles (Fig. 4c). Inclusion bodies and autophagosomes were infrequently observed in confluency-aged wild-type cultures (data not shown).

### Homozygous Cb*Cln3*^Δex7/8 ^cells and *Cln3*^Δex7/8 ^knock-in mice process cathepsin D abnormally

**Figure 5:**
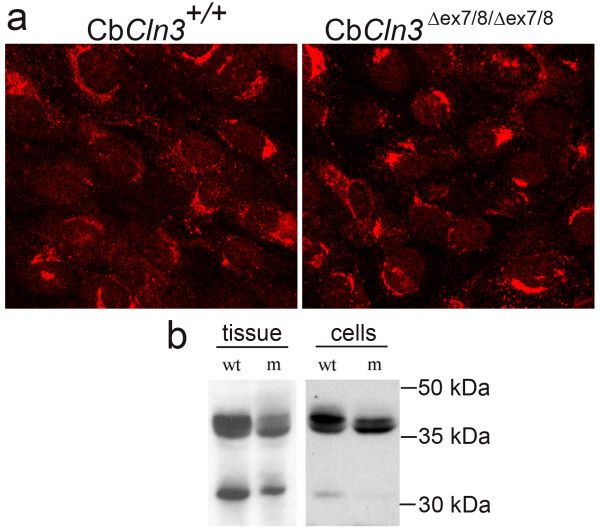
**Cathepsin D localization and processing in wild-type and homozygous Cb*Cln3*^Δex7/8 ^cells **a. Immunostaining of wild-type and homozygous Cb*Cln3*^Δex7/8 ^precursor cells with anti-cathepsin D antibody, recognizing unprocessed and processed forms of cathepsin D protein is shown. Cb*Cln3*^+/+ ^cells (left panel) exhibited a perinuclear and cytoplasmic punctate signal. Cathepsin D signal in homozygous Cb*Cln3*^Δex7/8 ^cells (right panel) was more often perinuclear, with less cytoplasmic punctate signal, compared to wild-type Cb*Cln3*^+/+ ^cells. 40 × magnification. b. α-Cathepsin D-probed immunoblots of total wild-type versus homozygous *Cln3*^Δex7/8 ^knock-in tissue or Cb*Cln3*^Δex7/8 ^cellular extracts are shown. The ~45 kDa cathepsin D band, representing precursor, was the predominant band in wild-type (wt) tissue and cellular extracts, with lower levels of mature enzyme (single chain, ~43 kDa, and heavy chain, ~31 kDa). Conversely, homozygous *Cln3*^Δex7/8 ^and Cb*Cln3*^Δex7/8 ^mutant (m) extracts exhibited reduced levels of precursor and heavy chain of the double-chain form of the enzyme, with elevated levels of single-chain mature enzyme.

We next investigated the basis for subunit c accumulation, testing the hypothesis that cathepsin D is abnormal since it is required for ATP synthase subunit c degradation in the lysosome [13]. We first tested cathepsin D transport and processing in homozygous Cb*Cln3*^Δex7/8 ^cells and *Cln3*^Δex7/8 ^mice using anti-cathepsin D antibody that recognizes unprocessed and processed cathepsin D isoforms. Immunostaining of wild-type and homozygous Cb*Cln3*^Δex7/8 ^cells revealed a perinuclear and punctate vesicular cathepsin D distribution, consistent with its transport and processing through the secretory pathway and delivery to the lysosome (Fig. 5a). However, in homozygous Cb*Cln3*^Δex7/8 ^cells, cathepsin D distribution was less vesicular and more perinuclear-clustered than in wild-type cells. Immunoblots of homozygous Cb*Cln3*^Δex7/8 ^cell and *Cln3*^Δex7/8 ^tissue extracts also showed altered relative levels of cathepsin D isoforms (Fig. 5b). Cathepsin D isoforms, identified by relative molecular weights, represent the ~45 kDa precursor, the ~43 kDa intermediate single chain form of the enzyme, and the 31 kDa heavy chain of the double-chain form of the mature enzyme [14]. In homozygous Cb*Cln3*^Δex7/8 ^cell and *Cln3*^Δex7/8 ^tissue extracts, the precursor and heavy chains were reduced, and the single chain was slightly elevated compared to wild-type extracts. The cellular growth media did not contain altered levels of cathepsin D, indicating enzyme secretion was not affected. Heterozygous *Cln3*^Δex7/8 ^mice and Cb*Cln3*^Δex7/8 ^cells were indistinguishable from wild-type, as expected for a recessive disease phenotype (data not shown).

The impact of the altered cathepsin D processing on enzymatic activity was next tested to determine if altered enzymatic activity accounts for inefficient subunit c turnover. In a fluorogenic *in vitro *assay, cathepsin D activity in total cellular extracts was not significantly altered in homozygous Cb*Cln3*^Δex7/8 ^cells (376 ± 89 RFU/μg total protein), versus wild-type cells (324 ± 58 RFU/μg total protein), although a consistent trend towards increased enzymatic activity in mutant cells was observed. Thus, cathepsin D transport and processing are disrupted in homozygous Cb*Cln3*^Δex7/8 ^cells in a manner such that enzymatic activity appears to be relatively unaffected.

### Homozygous Cb*Cln3*^Δex7/8 ^cells show abnormal membrane organelles

**Figure 6:**
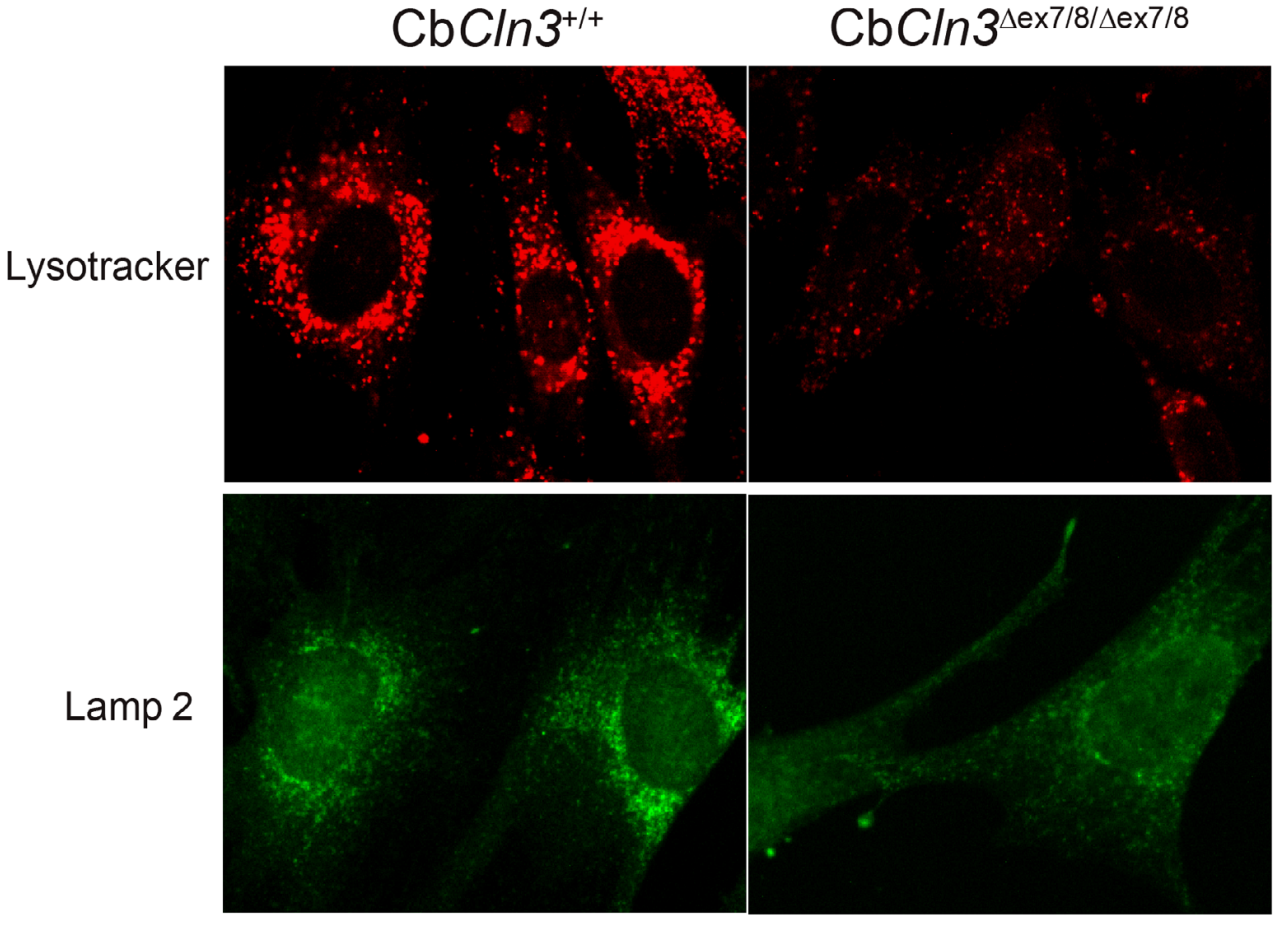
**Lysotracker and Lamp 2 labeling of wild-type and homozygous Cb*Cln3*^Δex7/8 ^lysosomes **Lysosomal labeling of wild-type and homozygous Cb*Cln3*^Δex7/8 ^precursor cells with lysotracker and Lamp 2 antibody is shown. Lysotracker dye (top panels) labeled large, perinuclear-clustered lysosomes and scattered lysosomes in the periphery of wild-type cells (Cb*Cln3*^+/+^). Lysotracker stain was dramatically reduced in homozygous mutant cells (Cb*Cln3*^Δex7/8/Δex7/8^), with smaller labeled vesicles and less apparent perinuclear clustering. Lamp 2 (bottom panels) immunostaining also showed reduced signal intensity with less perinuclear clustering in homozygous Cb*Cln3*^Δex7/8 ^cells, although the effect was somewhat less dramatic than that observed with Lysotracker dye. Wild-type and homozygous Cb*Cln3*^Δex7/8 ^confocal images were captured with identical exposure settings. 60 × magnification.

The abnormal transport and processing of cathepsin D suggested membrane trafficking disruptions in homozygous Cb*Cln3*^Δex7/8 ^cells; therefore, we surveyed the subcellular distribution and morphology of membrane organelles. Components of the secretory pathway, including the ER, *cis*-Golgi, and *trans*-Golgi, did not appear altered from wild-type appearance when labeled with the respective markers, protein disulfide isomerase (PDI), GM130, and VVL (data not shown). By contrast, the lysosomal markers, Lysotracker and Lamp 2 had significantly altered signal in homozygous Cb*Cln3*^Δex7/8 ^cells, versus wild-type cells. Wild-type cells exhibited brightly stained lysosomes that were large and clustered in the perinuclear region whereas homozygous Cb*Cln3*^Δex7/8 ^lysosomes were lightly stained, smaller vesicles that were more diffusely scattered in the cytoplasm of the cell (Fig. 6). Lamp 1 distribution was also altered, as previously noted (Fig. 3). However, Lamp 1 and Lamp 2 total protein levels were similar in wild-type and homozygous Cb*Cln3*^Δex7/8 ^cells by immunoblot analysis, indicating the altered signal likely reflects dispersed lysosomes or altered localization and/or epitope availability (data not shown). It is noteworthy that Lysotracker dye, which selectively accumulates in acidic compartments, exhibited the most marked reduction in lysosomal labeling. This observation may reflect altered lysosomal pH, an established finding in JNCL [10,15].

**Figure 7:**
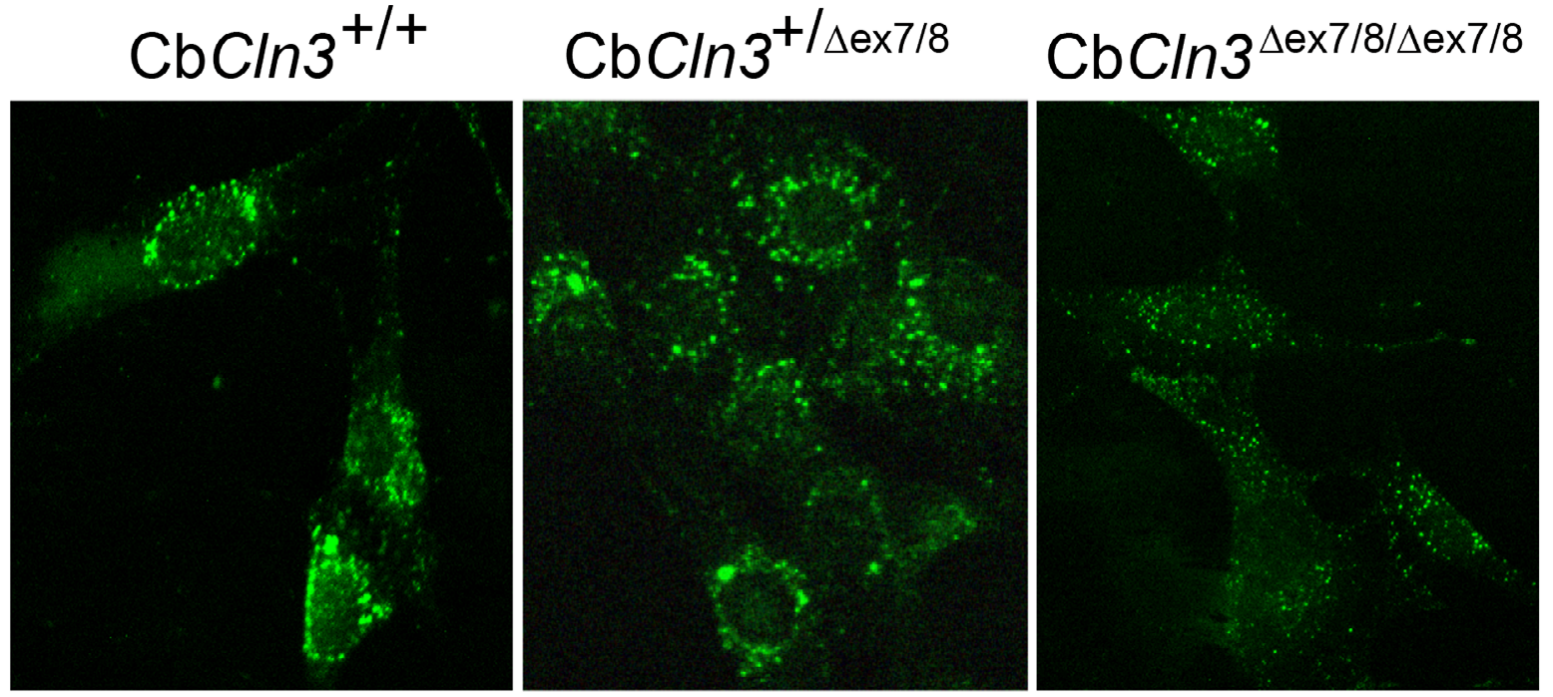
**Endocytosis in wild-type, heterozygous and homozygous Cb*Cln3*^Δex7/8 ^cells **Dextran-FITC uptake in wild-type, heterozygous and homozygous Cb*Cln3*^Δex7/8 ^precursor cells is shown. In wild-type (Cb*Cln3*^+/+^, left panel) and heterozygous (Cb*Cln3*^+/Δex7/8^, middle panel) cells, dextran-FITC label was observed in a perinuclear-clustered vesicular pattern with scattered labeled vesicles also present in the periphery. In contrast, dextran-FITC label of homozygous mutant (Cb*Cln3*^Δex7/8/Δex7/8^, right panel) cells was reduced overall and exhibited smaller stained vesicles with less perinuclear clustering. Confocal images were captured with identical exposure settings. 40 × magnification.

Consistent with the altered early endosome marker (EEA1) signal observed by immunostaining (Fig. 3), fluid-phase endocytosis was also altered in homozygous Cb*Cln3*^Δex7/8 ^cells, as measured by dextran-FITC uptake (Fig. 7). Following a 15-minute incubation in media containing dextran-FITC, wild-type and heterozygote cells displayed brightly stained, large endocytic vesicles that were clustered in the perinuclear region. However, homozygous Cb*Cln3*^Δex7/8 ^cells were less brightly stained with most dextran-FITC signal localizing to smaller vesicles scattered throughout the cytoplasm of the cell.

**Figure 8:**
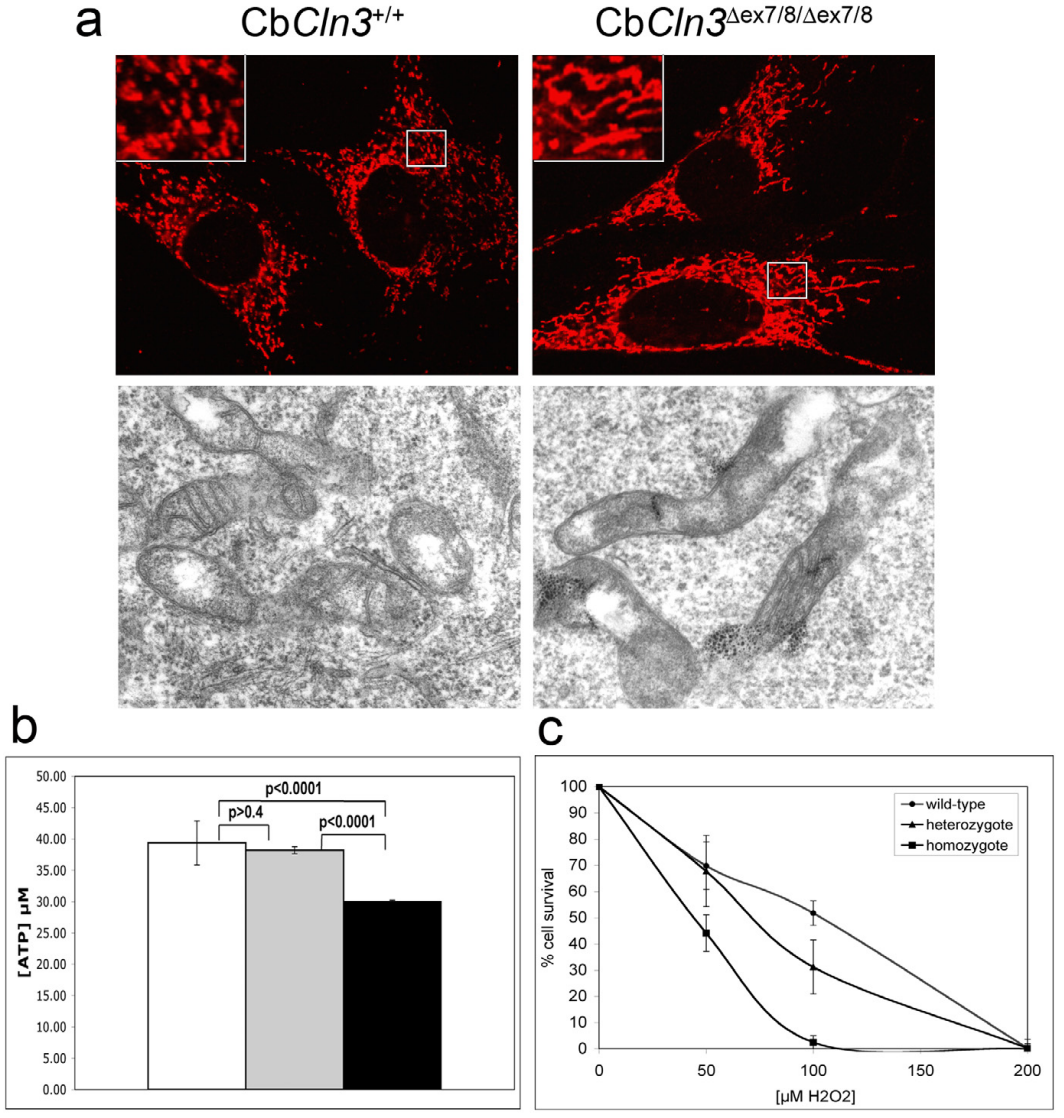
**Mitochondrial morphology and function in wild-type, heterozygous and homozygous Cb*Cln3*^Δex7/8 ^cells **a. Confocal and TEM micrographs of wild-type and homozygous Cb*Cln3*^Δex7/8 ^mitochondrial morphology are shown. Immunostaining with the inner mitochondrial membrane marker, grp75 (top panels) highlighted elongated mitochondria in homozygous mutant cells (Cb*Cln3*^Δex7/8/Δex7/8^), relative to wild-type mitochondria (Cb*Cln3*^+/+^) (insets, zoom = 2.75x). Mitochondrial distribution was not altered from the wild-type pattern. Elongated homozygous Cb*Cln3*^Δex7/8 ^mitochondria were also observed by TEM analysis. 60 × magnification. b. Cellular ATP levels in wild-type, heterozygous and homozygous Cb*Cln3*^Δex7/8 ^precursor cells are shown. Wild-type (open bar) and heterozygous (gray bar) Cb*Cln3*^Δex7/8 ^cells contained ~39 μM ATP, while homozygous Cb*Cln3*^Δex7/8 ^cells (black bar) contained ~1.3 fold reduced levels of ATP (~30 μM), which was statistically significant in a t-test (p < 0.0001). Wild-type and heterozygous Cb*Cln3*^Δex7/8 ^cellular ATP levels were not statistically different from each other (p > 0.4). A representative of triplicate experiments is shown (n = 6 in each experiment). c. Cell survival following 24-hour hydrogen peroxide treatment is shown. Homozygous Cb*Cln3*^Δex7/8 ^cells were ~2-fold more sensitive to oxidative stress by hydrogen peroxide treatment. Wild-type (circle) and heterozygous (triangle) Cb*Cln3*^Δex7/8 ^cells exhibited ~50% survival rates with 75–100 μM H_2_O_2_, whereas homozygous Cb*Cln3*^Δex7/8 ^cells (squares) had a ~50% survival rate with 50 μM H_2_O_2_. A representative of triplicate experiments is shown (n = 4 in each experiment).

Finally, because subunit c is a mitochondrial protein and its turnover proceeds through autophagic engulfment of mitochondria [13], we analyzed homozygous Cb*Cln3*^Δex7/8 ^cell mitochondrial morphology and function. Mitochondrial distribution in homozygous Cb*Cln3*^Δex7/8 ^cells was indistinguishable from wild-type and heterozygous cells; however, homozygous Cb*Cln3*^Δex7/8 ^mitochondria appeared more elongated by grp75 marker immunostaining and TEM analysis (Fig. 8a). 72% of homozygous mutant mitochondria were greater than 0.6 μm in length (range = 0.26 μm to 2.75 μm), while fewer wild-type mitochondria (51%) reached this length (range = 0.15 μm to 2.29 μm). Mitochondrial width was not altered in homozygous Cb*Cln3*^Δex7/8 ^cells (data not shown). Moreover, compared to wild-type or heterozygous cells, homozygous Cb*Cln3*^Δex7/8 ^cells had significantly reduced cellular ATP levels (1.3 fold less, Fig. 8b) and exhibited reduced survival following hydrogen peroxide treatment (~50% of wild-type survival, Fig. 8c), suggesting impaired energy metabolism and oxidative stress response. Taken together, these data support impaired mitochondrial function in homozygous Cb*Cln3*^Δex7/8 ^cells.

## Discussion

Cb*Cln3*^Δex7/8 ^cerebellar precursor cells represent the first genetically accurate neuron-derived culture model of JNCL. Homozygous Cb*Cln3*^Δex7/8 ^cells express mutant battenin and JNCL-hallmark mitochondrial ATPase subunit c accumulation, upon aging of cells at confluency. Moreover, this is the first study to indicate recessive endosomal/lysosomal membrane trafficking defects and mitochondrial dysfunction that precedes subunit c deposition in an accurate JNCL model. Importantly, these defects are likely to be early events in the JNCL disease process and may particularly impact neuronal function.

Abnormal cathepsin D localization and processing in homozygous Cb*Cln3*^Δex7/8 ^cells and *Cln3*^Δex7/8 ^mice likely reflects altered vesicular trafficking and/or lysosomal pH, which is known to impact cathepsin D processing [14,16]. Indeed, *CLN3 *overexpression in HEK-293 cells altered lysosomal pH and cathepsin D processing [17], and lysosomal pH homeostasis is disrupted in JNCL [10,15]. It is noteworthy that cathepsin B and the CLN2-encoded enzyme, TPPI, are also altered in JNCL [18-20]. Nevertheless, despite the cathepsin D protein alterations that are observed in homozygous Cb*Cln3*^Δex7/8 ^cells, cathepsin D enzymatic activity does not appear to be reduced. Thus, decreased cathepsin D activity is unlikely to account for subunit c accumulation in JNCL.

Aging of homozygous Cb*Cln3*^Δex7/8 ^cells at confluency is necessary to induce significantly accumulated subunit c protein. However, membrane organelle disruptions precede subunit c accumulation in homozygous Cb*Cln3*^Δex7/8 ^cells, since they are observed without aging at confluency. Lysosomal and endosomal size and distribution are altered, and mitochondria are abnormally elongated and functionally compromised in sub-confluent homozygous Cb*Cln3*^Δex7/8 ^cultures. These observations argue that membrane trafficking defects do not result from excessive subunit c accumulation compromising the lysosome, but rather are early events in the disease process preceding subunit c accumulation. Mitochondrial abnormalities, which have also been reported in JNCL patients and other animal models [21-23], may result from ineffective turnover by autophagy, a lysosomal-targeted pathway [24]. Alternatively, or simultaneously, battenin deficiency may impact mitochondrial function upstream of turnover, affecting mitochondrial biogenesis and/or altered transport and processing of mitochondrial proteins.

In wild-type Cb*Cln3 *neuronal precursor cells battenin primarily co-localizes with early and late endosomes. Battenin immunostaining in homozygous Cb*Cln3*^Δex7/8 ^neuronal precursors is significantly reduced in abundance, but mutant signal also co-localizes with endosomal markers suggesting mutant battenin protein with C-terminal epitopes is trafficked similar to wild-type protein. In other studies, CLN3/battenin protein localization has been reported to partially overlap with late endosomes and lysosomes in non-neuronal cells [7], and to lysosomes, synaptosomes [8] and endosomes [9,25] in neurons. These data jointly indicate that battenin resides in a subset of vesicular compartments linking multiple membrane trafficking pathways, perhaps functioning in vesicular transport and/or fusion. Endocytic and lysosomal-targeted pathways, including mitochondrial autophagy, are specifically implicated in this study.

## Conclusions

The membrane trafficking and mitochondrial deficits uncovered in homozygous Cb*Cln3*^Δex7/8 ^cells are likely to particularly impact neuronal function. Neurotransmission heavily relies on membrane vesicle transport, and a high-energy metabolism may further sensitize neurons to the loss of battenin activity. Thus, our panel of wild-type, heterozygous, and homozygous Cb*Cln3*^Δex7/8 ^cerebellar cells provide an ideal model system to further elucidate battenin function and JNCL pathogenesis.

## Methods

### Antibody and cell staining reagents

Nestin (clone Rat 401, 2 μg/ml), Lamp 1 (clone 1D4B, 6 μg/ml), and Lamp 2 (clone Abl-93; 6 μg/ml) monoclonal antibodies were obtained from the Developmental Studies Hybridoma Bank, maintained by The University of Iowa, Department of Biological Sciences. Batp1 (1 μg/ml) was previously described [12]. Anti-subunit c antibody (0.7 μg/ml) was kindly provided by Dr. Kominami (Juntendo University, Tokyo, Japan). Additional antibodies were as follows: GFAP, 1:2000 (DAKO Corporation); calbindin, 1:5000 (Sigma); NeuN, 10 μg/ml (Chemicon); SV40 T antigen (Pab 101), 2 μg/ml (Santa Cruz Biotechnology); cathepsin D (C-20), 2 μg/ml (Santa Cruz Biotechnology); cytochrome c oxidase subunit IV (cox4), 1:1000 (BD Biosciences Clontech); PDI (H-160), 2 μg/ml (Santa Cruz Biotechnology); GM130, 1 μg/ml (BD Transduction Labs); α-tubulin, 1:15,000 (Sigma); grp75, 1:200 (Stressgen); early endosome antigen-1 (EEA1), 2 μg/ml (Santa Cruz Biotechnology); rab 7, 4 μg/ml (Santa Cruz Biotechnology). All fluorescent secondary antibodies were obtained from Jackson ImmunoResearch Laboratories and HRP-conjugated secondary antibodies were obtained from Amersham Biosciences. Additional cell markers were as follows: VVL-biotin, 1:2000 (Vector Laboratories), 10,000 MW dextran-FITC, 1 mg/ml and Lysotracker Red DND-99, 500 nM (Molecular Probes).

### Generation, maintenance and differentiation of Cb*Cln3 *cerebellar neuronal precursor lines

*Cln3*^Δex7/8 ^knock-in mice have been previously described [12]. Littermate pups from heterozygote crosses were used for primary culture establishment, by previously described methods that yield cerebellar granule neuron-enriched cultures [26]. Postnatal day 4 (P4) cerebella were dissected in Hank's Balanced Salt Solution (HBSS, Sigma), supplemented with 35 mM glucose. Tail biopsies were also collected for genomic DNA isolation and genotypic analysis. Trypsin/EDTA (10 mg/ml, Sigma) and DNase I (100 μg/ml, Sigma), suspended in HBSS, helped dissociate cerebella for primary culture plating onto 0.01% poly-ornithine (Sigma) coated 100 mm dishes. Primary cultures from individual cerebella were cultured overnight at 37°C, 5% CO_2_, in granule neuron growth media (Dulbecco's Modified Eagle Medium [DMEM, Gibco BRL #11995-065], 10% fetal bovine serum [Sigma #F-2442], supplemented with 24 mM KCl). Infection was performed the following day with defective retrovirus transducing the temperature-sensitive tsA58/U19 large T antigen and a selection neomycin-resistance cassette [27], as previously described [28]. Following infection, cultures were shifted to the tsA58/U19 permissive growth temperature of 33°C and selection was in the same growth media as above, with 200 μg/ml G418. Conditionally immortalized colonies were isolated 3–9 weeks post-infection and expanded for frozen stocks and further sub-clone isolation. Multiple clonal lines were obtained for each genotype and all phenotypes were confirmed in at least 2 independent Cb*Cln3 *cell lines. Cb*Cln3 *cell lines were maintained on 0.01% poly-ornithine coated dishes at 30–90% confluency, in 33°C and 5% CO_2 _atmosphere. Passage number was recorded (up to ~20 passages), but had no apparent impact on phenotype. Neuronal differentiation was as previously described [29] with the following cocktail: 10 ng/ml α-FGF, 250 μM IBMX, 200 nM TPA, 50 μM forskolin, 5 μM dopamine (Sigma).

### Genotyping and RT-PCR

Genomic DNA was extracted from tail biopsies and cell pellets as described (Cotman et al., 2002). *Cln3*^Δex7/8 ^knock-in allele PCR genotyping was with wild-type primers, WtF (5'-CAGCATCTCCTCAGGGCTA-3') and WtR (5'-CCAACATAGAAAGTAGGGTGTGC-3') to yield a ~250 bp band and knock-in primers, 552F (5'-GAGCTTTGTTCTGGTTGCCTTC-3') and Ex9RA (5'-GCAGTCTCTGCCTCGTTTTCT-3') to yield a ~500 bp band. PCR cycling conditions were 95°C for 30 seconds, 58°C for 30 seconds, and 72°C for 35 seconds, repeated for 34 cycles. Total RNA isolation and *Cln3 *RT-PCR primers and procedures have been previously described [12].

### Subunit c accumulation assay

Cells were seeded into 4-well chamber-slides (Falcon) at a density of 5 × 10^4 ^cells per well for microscopy studies, or into 100 mm dishes (Falcon) at a density of 5 × 10^5 ^cells per dish for protein extraction. Cells were typically >95% confluent one day post-plating, and the following day was considered 1-day post-confluency. At the indicated times, cells were either fixed with 4% formaldehyde in phosphate buffered saline (PBS), pH 7.4, for 20 minutes and processed for autofluorescence/subunit c immunostaining, or cell pellets were collected for total protein extraction.

Alternatively, cells were fixed with 2.5% glutaraldehyde/2% paraformaldehyde in 0.1 M cacodylate buffer, pH 7.4 for 1 hour and subsequently post-fixed and processed for TEM analysis as described [12]. In confocal microscopy studies, autofluorescent signal was observed over multiple wavelengths. For co-staining, settings were reduced such that autofluorescent signal did not contribute to antibody label signal.

### Immunostaining and Immunoblot analysis

For immunostaining, cells were seeded at a density of 3–5 × 10^4 ^cells per well in 4-well chamber-slides and grown overnight at 33°C, unless indicated otherwise. Fixation was with ice-cold 4% formaldehyde in PBS, pH 7.4, for 20 minutes, or with ice-cold methanol/acetone (1:1) for 10 minutes at -20°C followed by air-drying (antibody-dependent). Cells were washed with PBS at least 2 times, 5 minutes per wash, between each of the following steps of the staining procedure: 0.1 M glycine in PBS for 5 minutes, 0.05% or 0.1% (antibody-dependent) Triton X-100 (Fisher Scientific) in PBS for 5 minutes, 2% bovine serum albumin (BSA) in PBS for 30 minutes, primary antibody diluted in 2% BSA/PBS for 90 minutes, secondary antibody diluted in 2% BSA/PBS for 60 minutes. All incubations were carried out at room temperature. Following staining procedures, slides were coverslipped with Vectashield mounting medium (Vector Laboratories) and analyzed on a BioRad Radiance 2100 confocal microscope (Biorad), with identical exposure settings for wild-type and mutant like images. All comparisons of wild-type and mutant staining were performed in Adobe Photoshop with identical brightness and contrast adjustments.

Total proteins were isolated from cell pellets by extraction with ice-cold 20 mM Tris, pH 7.4, 1% Triton X-100 (membrane-research grade, Roche), plus protease inhibitors (Complete mini tablet, 0.7 μg/ml pepstatin A, 2 μg/ml aprotinin, 5 μg/ml leupeptin [Roche]). Following homogenization through a 25-gauge needle (~10 passes), extracts were centrifuged at 1000 × g for 10 minutes, at 4°C, to remove debris. Typically, 20–40 μg of protein (determined by Bio-rad D_c _Protein Assay) was separated by SDS-PAGE, for subsequent immunoblotting, as described [12]. 16.5% tris-tricine SDS-PAGE gels were used for subunit c immunoblotting experiments.

### Cathepsin D activity assay

100 mm tissue culture dishes, which were approximately 80–90% confluent, were washed briefly with ice-cold PBS, and total protein extracts were isolated by scraping cells into 10 mM Tris, pH 7.4, 0.1% Triton X-100 followed by incubation on ice, for 20 minutes. The insoluble material was centrifuged at 14,000 g, the supernatant was isolated, and protein concentration was determined using the Bio-rad D_c _Protein Assay. 50–70 μg of total protein extract were used to measure cathepsin D activity using the Fluorogenic Innozyme™ Cathepsin D Immunocapture Activity Assay Kit (EMD Biosciences) according to the manufacturer's recommendations. Relative fluorescence was measured using an Analyst AD plate reader (Molecular Devices) with the following filters and settings: excitation filter, 360-35; emission filter, 400-20, Flash lamp with 100 readings/well, 100 ms between readings, and 100,000 μs integration time.

### Lysosomal staining and endocytic uptake

Cells were seeded at a density of 3–5 × 10^4 ^cells per well in 4-well chamber-slides and grown overnight at 33°C. Growth media was exchanged for fresh, pre-warmed growth media containing 500 nM Lysotracker or 1 mg/ml dextran-FITC, and cells were incubated at 33°C for 45 minutes or 15 minutes, respectively. Following labeling, cells were immediately placed on ice and washed for 10 minutes in ice-cold dye-free media, and fixed with 4% formaldehyde in PBS, for 20 minutes on ice. Chambers were removed and slides were coverslipped with Vectashield mounting media for confocal microscopy analysis, as described above.

### Morphometric analysis of mitochondria

TEM photomicrographs (10,000 × – 40,000 × magnification) were taken from random grid fields. For length measurements, the longest side of each mitochondria was measured in centimeters, and along the length of the mitochondria, width measurements were taken every 2.5–4 mm (dependent on the magnification of the micrograph image). Following measurement, all numbers were normalized to reflect one magnification and data was analyzed using Microsoft Excel software. To ascertain unmagnified mitochondrial size, final measurement data, in centimeters, was converted to nanometers according to scale bar representation.

### ATP measurement

ATP was measured by using the CellTiter-GLO^® ^Luminescent Cell Viability kit (Promega), according to the manufacturer's recommendations. Briefly, cells were plated in a black opaque-walled 96 well plate (Packard Bioscience) at a density of 20000/well and incubated at 33°C overnight. The following day, CellTiter-GLO^® ^Reagent was added to each well and cell lysis was induced by mixing 2 minutes. An ATP standard curve was prepared in the same plate. Before recording luminescence with a microplate luminometer (MicroLumat Plus LB 96V, Berthold Techonologies), the plate was dark adapted for 10 minutes at room temperature to stabilize the luminescence signal.

### Hydrogen peroxide treatment assay

Cells were plated at a density of 10,000 cells/well in 96-well plates and incubated at 33°C overnight. The following day, fresh media containing varying concentrations of hydrogen peroxide was dispersed to each well. Cells were incubated in the presence of hydrogen peroxide for 24 hours, at 33°C, and viability was measured using the CellTiter-96^® ^AQueous Non-Radioactive Cell Proliferation Assay (Promega), according to the manufacturer's specifications.

## Authors' contributions

EF participated in establishment and characterization of cell lines and performed ATP determinations. PW participated in mitochondrial analysis and immunocytochemistry. JE, TL-N, AMT, and HG participated in genotypic and additional phenotypic analysis of cell lines. DR and EC generated virus-conditioned medium for conditional immortalization of cells. MEM co-conceived of the study and assisted on drafting of the manuscript. SLC co-conceived of the study, participated in establishment and phenotypic analysis of cell lines, and drafted the manuscript. All authors read and approved the final manuscript.

## References

[B1] Rider JA, Rider DL (1988). Batten disease: past, present, and future. Am J Med Genet Suppl.

[B2] International Batten Disease Consortium (1995). Isolation of a novel gene underlying Batten disease, CLN3. The International Batten Disease Consortium. Cell.

[B3] Jolly RD, Martinus RD, Palmer DN (1992). Sheep and other animals with ceroid-lipofuscinoses: their relevance to Batten disease. Am J Med Genet.

[B4] Palmer DN, Fearnley IM, Walker JE, Hall NA, Lake BD, Wolfe LS, Haltia M, Martinus RD, Jolly RD (1992). Mitochondrial ATP synthase subunit c storage in the ceroid-lipofuscinoses (Batten disease). Am J Med Genet.

[B5] Wisniewski KE, Rapin I, Heaney-Kieras J (1988). Clinico-pathological variability in the childhood neuronal ceroid-lipofuscinoses and new observations on glycoprotein abnormalities. American Journal of Medical Genetics - Supplement.

[B6] Mao Q, Foster BJ, Xia H, Davidson BL (2003). Membrane topology of CLN3, the protein underlying Batten disease. FEBS Letters.

[B7] Ezaki J, Takeda-Ezaki M, Koike M, Ohsawa Y, Taka H, Mineki R, Murayama K, Uchiyama Y, Ueno T, Kominami E (2003). Characterization of Cln3p, the gene product responsible for juvenile neuronal ceroid lipofuscinosis, as a lysosomal integral membrane glycoprotein. Journal of Neurochemistry.

[B8] Luiro K, Kopra O, Lehtovirta M, Jalanko A (2001). CLN3 protein is targeted to neuronal synapses but excluded from synaptic vesicles: new clues to Batten disease. Human Molecular Genetics.

[B9] Kyttala A, Ihrke G, Vesa J, Schell MJ, Luzio JP (2004). Two motifs target Batten disease protein CLN3 to lysosomes in transfectedd nonneuronal and neuronal cells. Molecular Biology of the Cell.

[B10] Pearce DA, Ferea T, Nosel SA, Das B, Sherman F (1999). Action of BTN1, the yeast orthologue of the gene mutated in Batten disease. Nat Genet.

[B11] Kim Y, Ramirez-Montealegre D, Pearce DA (2003). A role in vacuolar arginine transport for yeast Btn1p and for human CLN3, the protein defective in Batten disease. Proceedings of the National Academy of Sciences of the United States of America.

[B12] Cotman SL, Vrbanac V, Lebel LA, Lee RL, Johnson KA, Donahue LR, Teed AM, Antonellis K, Bronson RT, Lerner TJ, MacDonald ME (2002). Cln3∆ex7/8 knock-in mice with the common JNCL mutation exhibit progressive neurologic disease that begins before birth. Human Molecular Genetics.

[B13] Kominami AE (2002). What are the requirements for lysosomal degradation of subunit c of mitochondrial ATPase?. IUBMB Life.

[B14] Partanen S, Storch S, Loffler HG, Hasilik A, Tyynela J, Braulke T (2003). A replacement of the active-site aspartic acid residue 293 in mouse cathepsin D affects its intracellular stability, processing and transport in HEK-293 cells. Biochemical Journal.

[B15] Holopainen JM, Saarikoski J, Kinnunen PK, Jarvela I (2001). Elevated lysosomal pH in neuronal ceroid lipofuscinoses (NCLs). European Journal of Biochemistry.

[B16] Nishimura Y, Kato K, Furuno K, Himeno M (1995). Biosynthesis and processing of lysosomal cathepsin D in primary cultures of rat hepatocytes. Biological & Pharmaceutical Bulletin.

[B17] Golabek AA, Kida E, Walus M, Kaczmarski W, Michalewski M, Wisniewski KE (2000). CLN3 protein regulates lysosomal pH and alters intracellular processing of Alzheimer's amyloid-beta protein precursor and cathepsin D in human cells. Mol Genet Metab.

[B18] Dawson G, Glaser PT (1988). Abnormal cathepsin B activity in Batten disease. Am J Med Genet Suppl.

[B19] Wisniewski KE, Kida E, Walus M, Wujek P, Kaczmarski W, Golabek AA (2001). Tripeptidyl-peptidase I in neuronal ceroid lipofuscinoses and other lysosomal storage disorders. European Journal of Paediatric Neurology.

[B20] Mitchison HM, Bernard DJ, Greene ND, Cooper JD, Junaid MA, Pullarkat RK, de Vos N, Breuning MH, Owens JW, Mobley WC, Gardiner RM, Lake BD, Taschner PE, Nussbaum RL (1999). Targeted disruption of the Cln3 gene provides a mouse model for Batten disease. The Batten Mouse Model Consortium [corrected]. [erratum appears in Neurobiol Dis 2000 Apr;7(2):127]. Neurobiol Dis.

[B21] March PA, Wurzelmann S, Walkley SU (1995). Morphological alterations in neocortical and cerebellar GABAergic neurons in a canine model of juvenile Batten disease. Am J Med Genet.

[B22] Das AM, Jolly RD, Kohlschutter A (1999). Anomalies of mitochondrial ATP synthase regulation in four different types of neuronal ceroid lipofuscinosis. Mol Genet Metab.

[B23] Das AM, von Harlem R, Feist M, Lucke T, Kohlschutter A (2001). Altered levels of high-energy phosphate compounds in fibroblasts from different forms of neuronal ceroid lipofuscinoses: further evidence for mitochondrial involvement. European Journal of Paediatric Neurology.

[B24] Klionsky DJ, Emr SD (2000). Autophagy as a regulated pathway of cellular degradation. Science.

[B25] Persaud-Sawin DA, McNamara JOII, Vandongen RS, Boustany R (2004). A galactosylceramide binding domain is involved in trafficking of CLN3 from golgi to rafts via recycling endosomes.. Pediatric Research.

[B26] D'Mello SR, Galli C, Ciotti T, Calissano P (1993). Induction of apoptosis in cerebellar granule neurons by low potassium: inhibition of death by insulin-like growth factor I and cAMP. Proceedings of the National Academy of Sciences of the United States of America.

[B27] Jat PS, Cepko CL, Mulligan RC, Sharp PA (1986). Recombinant retroviruses encoding simian virus 40 large T antigen and polyomavirus large and middle T antigens. Molecular & Cellular Biology.

[B28] Cattaneo E, Conti L (1998). Generation and characterization of embryonic striatal conditionally immortalized ST14A cells. Journal of Neuroscience Research.

[B29] Trettel F, Rigamonti D, Hilditch-Maguire P, Wheeler VC, Sharp AH, Persichetti F, Cattaneo E, MacDonald ME (2000). Dominant phenotypes produced by the HD mutation in STHdh(Q111) striatal cells. Hum Mol Genet.

